# The Relationship of Lifestyle with Disease Activity among Patients with Systemic Lupus Erythematosus: A Descriptive-Correlational Study

**DOI:** 10.31138/mjr.32.2.124

**Published:** 2021-06-30

**Authors:** Leila Sayadi, Seyedeh Tahereh Faezi, Marzieh Hasanpour, Sofia Jami Alahmadi

**Affiliations:** 1 Nursing and Midwifery Care Research Centre, School of Nursing & Midwifery,Tehran University of Medical Sciences, Tehran, Iran; 2 Rheumatology Research Centre, Tehran University of Medical Sciences, Tehran, Iran; 3 School of Nursing & Midwifery,Tehran University of Medical Sciences, Tehran, Iran

**Keywords:** Systemic lupus erythematosus, lifestyle, disease activity

## Abstract

**Objective:**

The objective of this study was to evaluate the relationship of lifestyle with disease activity among patients with systemic lupus erythematosus.

**Methods:**

This cross-sectional descriptive-correlational study was conducted in 2019 on 209 patients with systemic lupus erythematosus. Data were collected using a demographic and clinical characteristics questionnaire, the Health-Promoting Lifestyle Profile II, and the systemic lupus erythematosus disease activity Index. The data were analysed through the mixed model and the logistic regression analyses.

**Results:**

In total, 67.5% of participants had active disease. The mean score of lifestyle was 2.49±0.30 and the lowest and the highest lifestyle dimensional mean scores were respectively related to the physical activity and the health responsibility dimensions (1.55±0.60 and 3.25±0.45). The analysis showed that each one point increase in the mean score of lifestyle was associated with 0.79 point decrease in the odds of disease activity (P = 0.006). Moreover, disease activity had significant positive relationship with body mass index (P = 0.015).

**Conclusion:**

Interventions for promoting lifestyle among these patients and improving healthcare providers’ knowledge about Systemic lupus erythematosus and lifestyle modification are recommended to reduce disease activity.

## INTRODUCTION

Systemic lupus erythematosus (SLE) is a chronic multisystemic autoimmune disease. Its pathogenesis is unknown, although genetic predisposition and environmental factors such as solar ultraviolet radiation, infections, and medications may trigger it. In SLE, antibodies react to normal body cells instead of foreign antigens, resulting in the formation of immune complexes which deposit in different body tissues and cause tissue injury.^1^ The incidence of SLE is 1–10 cases per 100000 person-years, and its prevalence is 20–200 cases per 100000.^2,3^ A study in Iran showed that one per 2500 Iranians is afflicted by SLE.^4,5^ SLE is prevalent in ages between 14 and 44 years, and 90% of patients with SLE are women.^2,6^

SLE is associated with many different symptoms and consequences. Its symptoms include skin rashes, photophobia, joint swelling and pain, weakness, fatigue, and kidney disorders.^2^ Multisystemic involvement causes patients different problems, imposes financial strains on them due to treatment costs and disability, and makes them dependent and unable to fulfil their care-related needs.^7^ SLE considerably restricts daily activities, particularly at the time of pain recurrence, and causes difficulties in employment, interpersonal relationships, and social roles.^8^ Repeated relapses and progression of SLE considerably affect the musculoskeletal system, skin, kidneys, heart, lungs, and central nervous system,^9^ resulting in frequent hospitalisations. SLE treatment includes immunosuppressive agents, which are in turn associated with many different side effects and complications.^10^ SLE-related mortality is related to disease activation, infections, nephritis, acute renal failure, thrombosis secondary to antiphospholipid syndrome, carditis, pneumonia, pulmonary hypertension, cardiovascular complications, atherosclerosis, stroke, and myocardial infarction. These factors increase SLE-related mortality 2 or 5 times greater than the mortality rate in the general population.^9,11^

SLE is associated with courses of relapse and remission. In other words, it may relapse due to many different factors, even in case of complete recovery with treatments. SLE relapse and activation are associated with a course of active inflammation throughout the body.^12^ Therefore, reducing disease activity (DA) is a main outcome in SLE treatment and care.^13^ SLE severity and DA are affected by a wide range of factors, including climate, genetic factors, race, ethnicity, and sociocultural status.^5,14–16^ Lifestyle is also a significant factor that contributes to SLE relapse and DA.

Lifestyle is defined as individuals’ beliefs about and their strategies for health-related behaviours, including nutrition, physical activity, health responsibility, stress management, interpersonal relationships, and spiritual growth.^16,17^ Lifestyle reflects attitudes, values, and self-image, and is reflected in daily activities, such as eating, rest and exercise, relationships, thinking, planning, driving, sleep, and work. It is greatly affected by culture, environment, personal characteristics, personal and social relationships,^18^ geographical environment, and ethnicity.^5,14–16^ Lifestyle greatly affects general health, and has a significant relationship with most health problems including chronic diseases.^19^

As SLE is a chronic disease with lifelong symptoms, lifelong need for treatment and care, frequent courses of relapse and remission, and considerable effects on different body systems, afflicted patients need to have lifelong adherence to a healthy lifestyle.^15,16,20–23^ These patients need to engage in healthy lifestyle behaviours, such as regular physical activity, relationships with family members and close friends, tobacco abstinence, and a diet high in polyunsaturated fatty acids.^16^ Nonetheless, previous studies reported poor lifestyle among these patients. For instance, a study in Iran showed that patients with SLE did not have a healthy dietary regimen.^14^ Another study in Taiwan reported that patients with SLE had poor lifestyle, 72% of them had low sleep quality, and 20%–32% of them suffered from severe depression and anxiety.^24^ Similarly, a study in Egypt showed that more than 75% of patients with SLE were overweight or obese.^25^

Lifestyle has potential effects on DA among patients with SLE. A study in Sweden showed that patients with SLE who had healthier lifestyle experienced lower levels of fatigue.^15^ Another study in Egypt reported that SLE DA had significant relationship with body mass index.^25^ A systematic review also showed that DA in SLE had a significant positive relationship with tobacco use.^16^

Although there are wide differences among different geographical areas respecting lifestyle behaviours and SLE severity and DA,^26^ limited studies have so far addressed the relationship of SLE DA with lifestyle in Iran. Most studies on patients with SLE in Iran were on SLE epidemiology, characteristics,^4,5^ diagnosis, treatment, and associated outcomes^27^ such as depression, anxiety, and quality of life.^8^ This study sought to address this gap. The aim of the study was to evaluate the relationship of lifestyle with DA among patients with SLE.

## MATERIALS AND METHODS

### Design

This cross-sectional descriptive-correlational study was conducted from April to December 2019.

### Setting and participants

**Figure 1.**
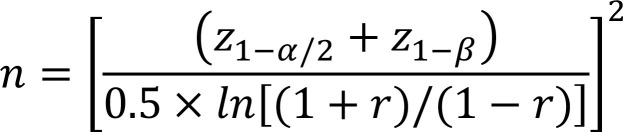
Sample size calculation.

Study setting was the rheumatology clinic of a large-scale public teaching hospital affiliated to Tehran University of Medical Sciences, Tehran, Iran. Study population consisted of all patients who referred to the study setting. Participants were 209 patients with SLE who were recruited through consecutive sampling. Inclusion criteria were age over eighteen years, ability to read and write in Persian, definite diagnosis of SLE by a rheumatologist, history of referring to the study setting for receiving SLE treatment and care for at least one year, and consent for participation. Participants were excluded if they refrained from answering the study instruments. With a correlation coefficient of 0.22 between lifestyle and quality of life,^24^ a type I error of 0.05, and a type II error of 0.10, sample size was estimated to be 209 (**Figure 1**).

### Instruments

Data were collected using a demographic and clinical characteristics questionnaire, the Health-Promoting Lifestyle Profile II (HPLPII), and systemic lupus erythematosus disease activity index (SLEDAI). The items of the demographic and clinical characteristics questionnaire were on age, gender, marital status, number of children, educational level, occupation, place of residence, living arrangement, positive family history of SLE, cigarette smoking, alcohol consumption, drug abuse, duration of affliction by SLE, body mass index, and history of hypertension and pulmonary, haematologic, renal, and hepatic disorders. HPLPII, developed based on Pender’s Health Promotion Model, contains 52 items scored 1 (“Never”), 2 (“Sometimes”), 3 (“Often”), or 4 (“Routinely”). The six dimensions of HPLPII are nutrition (nine items), physical activity (eight items), health responsibility (nine items), stress management (eight items), interpersonal relationships (nine items), and spiritual growth (nine items). The total score of HPLPII and each of the dimensions is obtained through the sum of the scores that ranged from 52 to 208. However, the use of the mean rather than the sum of the scales is recommended. So, the total score of each dimension is calculated through dividing its sum score by the number of its items and the total score of HPLPII is calculated through dividing the sum score of all 52 items by 52. Therefore, the score of HPLPII and its dimensions can range from 1 to 4.^28–30^ Previous studies reported acceptable reliability and validity for the Persian and Turkish HPLPII.^17,28,31^ Studies in Iran reported that the Cronbach’s alpha values of the dimensions of the Persian HPLPII were 0.79–0.87.^17,28^ In the present study, HPLPII validity was approved by 13 experts in nursing and rheumatology and its Cronbach’s alpha was 0.71. SLEDAI is an appropriate checklist for DA assessment among patients with SLE.^32^ It consists of 24 items, namely 16 items on clinical findings, and eight items on laboratory findings.^32^ The first eight items are on seizure, psychosis, organic brain syndrome, visual disturbance, lupus headache, cranial nerve disorder, cerebrovascular accident, and vasculitis, and are scored either 0 (“No”) or 8 (“Yes”). The next six items are on arthritis, myositis, urinary cast, haematuria, proteinuria, and pyuria and are scored 0 (“No”) or 4 (“Yes”). The next seven items are on skin rash, alopecia, oral or nasal mucosal ulcers, pleurisy, pericarditis, low complement, and increased DNA binding and are scored either 0 (“No”) or 2 (“Yes”). The last three items are on fever, thrombocytopenia, and leukopenia and are scored either 0 (“No”) or 1 (“Yes”). The possible total score of SLEDAI is 0–105. Scores less than 6 show inactive SLE, while scores 6 and greater show active SLE. This checklist assesses DA in the past 10 days and is sensitive to DA variations in the past 30 days.^22,33^

### Procedure

Participants completed the demographic and clinical characteristics questionnaire and HPLPII through the self-report method. Some participants felt tired at completing these instruments and hence, one of authors (SJ) completed these instruments for them through the interview method. Moreover, the original version of the SLEDAI checklist was completed for participants by rheumatologists based on participants’ clinical and laboratory characteristics.

### Statistical analysis

The STATA16 (StataCorp, College Station, Texas, USA) was used for data analysis. Categorical variables were described through the absolute and the relative frequency measures, while numerical variables were described through the mean and standard deviation measures. The normality of the data was assessed and confirmed through the skewness and the kurtosis measures. The mean scores of lifestyle dimensions were compared with each other through the mixed model analysis with compound symmetry covariance structure. Difference of demographic characteristics with HPLPII were measured by Pearson, Independent sample T test, and ANOVA. The univariate and the multivariate logistic regression analyses were employed to evaluate the relationship of the dichotomous variable of DA (categorised as active or inactive) with the scores of lifestyle, its dimensions, and its personal characteristics. In univariate logistic regression analysis, each independent variable was entered into an independent model. For multivariate logistic regression analysis, hierarchical modelling was used, in which the scores of lifestyle and its dimensions were entered into a single model in the first step, and then demographic and clinical characteristics were entered into the same model in the next step. Odds ratio with 95% confidence interval (95% CI) was calculated as effect size. The goodness of fit of the final logistic regression model was tested through the Hosmer-Lemeshow test. Moreover, the accuracy, sensitivity, and specificity measures were calculated for the model. The level of significance in all analyses was set at less than 0.05.

## RESULTS

The means of participants’ age and body mass index were 41.32±12.15 years and 25.69±4.64, respectively. Most of participants were female (91.9%), more than two-fifths of them had below-diploma education (43.1%), and around half of them were overweight or obese (49.3%). The length of suffering from SLE was 137.8±111.4 months, on average (**Table 1**).

**Table 1.** Participants’ demographic characteristics.

**Participants’ demographic characteristics**		**Mean±SD or N (%)**
Age (Years)		41.32±12.15

Gender	Male	17 (8.1)
	Female	192 (91.9)

Marital status	Single	53 (27)
	Married	156 (74)

Educational level	Below diploma	90 (43.1)
	Diploma	73 (34.9)
	University	46 (22)

Cigarette smoking	Yes	7 (3.3)
	No	202 (96.7)

Duration of cigarette smoking (Years)		18±10.59

Number of cigars smoked daily		12.62±11.56

Alcohol consumption	Yes	3 (1.4)
	No	206 (98.6)

Duration of alcohol consumption (Years)		14.6±13.61

Positive family history of SLE	Yes	13 (6.2)
	No	196 (93.8)

Duration of affliction by SLE(Month)		137.8 ±111.4

Body mass index		25.69±4.64

Body mass index	< 18.5 (Underweight)	7 (3.3)
	18.5–24.9 (Normal weight)	99 (47.4)
	25.0–29.9 (Overweight)	65 (31.1)
	30.0–34.9 (Class I obesity)	27 (12.9)
	35.0–39.9 (Class II obesity)	10 (4.8)
	≥ 40 (Class III obesity)	1 (0.5)

SLE was active among 67.5% of participants (141 out of 209 participants). The mean score of HPLPII was 2.49±0.30 (in the possible range of 1–4) (**Table 2**). The results of the mixed model analysis with compound symmetry covariance structure showed that there was at least one significant difference between the dimensions of lifestyle. The results of the Sidak post hoc test revealed that except for the difference between the nutrition and the interpersonal relationships dimensions (P=0.139), all other pairwise differences between lifestyle dimensions were statistically significant (P<0.05). The lowest and the highest dimensional mean scores were related to the physical activity (1.55±0.60) and the health responsibility (3.25±0.45) dimensions (**Table 2**).

**Table 2.** The mean scores of HPLPII and SLEDAI.

**Variables**		**Mean±SD**	**95% CI**	**Range**
HPLPII	Total	2.49±0.30		1.83–3.56
	Physical activity	1.55±0.60	1.46–1.63	1.00–3.63
	Stress management	1.99±0.43	1.93–2.05	1.13–3.75
	Health responsibility	3.25±0.45	3.19–3.31	1.67–3.89
	Nutrition	2.87±0.40	2.81–2.92	1.67–3.67
	Spiritual growth	2.35±0.50	2.29–2.42	1.11–4.00
	Interpersonal relationships	2.77±0.47	2.71–2.84	1.44–3.78

SLEDAI		7.69±4.57		0–20

CI: Confidence interval.

There was a significant difference between educational level and HPLPII, so that patients with academic level had better HPLPII score comparing to below diploma (P=0.02). However, there was not any differences between diploma with academic(P=0.053) and below diploma (P=0.212) (**Table 3**).

**Table 3.** The difference of total mean scores of HPLPII with Participants’ demographic characteristics.

**Participants’ demographic characteristics**		**Total mean scores of HPLPII**	
		****	
		**Mean ± SD**	**P Value**
Age (Years)^*^			0.182

Gender^†^	Male	2.45±0.28	0.659
	Female	2.49±0.30	

Marital status^†^	Single	2.54±0.30	0.097
	Married	2.46±0.29	

Educational level^‡^	Below diploma	2.43±0.25	0.009
	Diploma	2.49±0.32	
	University	2.60±0.32	

Cigarette smoking^†^	Yes	2.31±0.38	0.177
	No	2.49±0.29	

Duration of cigarette smoking (Years) ^*^			0.15

Number of cigars smoked daily^*^			0.221

Alcohol consumption^†^	Yes	2.49±0.30	0.889
	No	2.49±0.30	

Duration of alcohol consumption (Years) ^*^			0.958

Positive family history of SLE^†^	Yes	2.47±0.38	0.939
	No	2.49±0.29	

Duration of affliction by SLE(Month) ^*^			0.496

Body mass index^*^			0.994

* Pearson correlation;

† Independent sample T test;

‡ ANOVA.

The results of the univariate logistic regression analysis showed that the mean scores of lifestyle and its stress management and spiritual growth dimensions had significant negative relationships with SLE DA, so that each one point increase in the mean scores of lifestyle and its stress management and spiritual growth dimensions was associated with respectively 0.76, 0.63, and 0.57 point decrease in the mean score of SLE DA. Moreover, the results of the same analysis revealed that the mean score of body mass index had significant positive relationship with SLE DA, so that the odds of DA were seven times greater among those who were overweight or had first-or second-class obesity (**Table 4**).

**Table 4.** The results of the univariate logistic regression analysis to evaluate the relationship of SLEDAI with HPLPII and personal characteristics.

**Independent variables**		**Odds Ratio**	**95% CI**	**P value**
Lifestyle	Total	0.241	0.088–0.660	0.006
	Physical Activity	0.873	0.544–1.403	0.576
	Stress management	0.370	0.186–0.740	0.005
	Health responsibility	0.536	0.265–1.083	0.082
	Nutrition	0.501	0.232–1.079	0.077
	Spiritual growth	0.426	0.232–0.780	0.006
	Interpersonal relationships	0.654	0.349–1.226	0.186

Age		1.011	0.987, 1.036	0.363

Gender	Male*	1.000		
	Female	0.255	0.057–1.147	0.075

Educational level	Below diploma*	1.000		
	Diploma	1.260	0.639–2.482	0.504
	University	0.676	0.324–1.409	0.296

Cigarette smoking	Yes*	1.000		
	No	0.336	0.040–2.846	0.317

Duration of cigarette smoking (Years)		1.038	0.946, 1.139	0.432

Number of cigars smoked daily		1.180	0.882, 1.579	0.266

Duration of affliction by SLE(Month)		0.999	0.997, 1.002	0.835

Body mass index		1.392 #	0.994, 1.950	0.054

Body mass index	< 18.5 (Underweight)*	1.000		
	18.5–24.9 (Normal weight)	4.375	0.807–23.714	0.087
	25.0–29.9 (Overweight)	7.059	1.251–39.841	0.027
	30.0–34.9 (Class I obesity)	7.143	1.121–45.518	0.037
	35.0–39.9 (Class II obesity)	6.667	0.809–54.957	0.078

Positive family history of SLE	Yes	1.000		
	No	0.358	0.077–1.663	0.190

CI: Confidence interval;

# Trend effect;

* Reference category.

The Hosmer-Lemeshow test revealed the goodness of fit of the final hierarchical logistic regression model (P > 0.05). The accuracy, sensitivity, and specificity values of the model for predicting the active state of SLE were 76%, 94%, and 38%, respectively. The results of the multivariate logistic regression analysis illustrated a significant negative relationship between the mean score of lifestyle and SLE DA, so that each one point increase in the mean score of lifestyle was associated with 0.79 point decrease in the odds of SLE DA. Moreover, SLE DA had significant positive relationship with body mass index, so that the odds of SLE DA among those who were overweight, had first-class obesity, and had second-class obesity were greater by eight, ten and nine times, respectively. The trend test also showed a significant trend for the mean score of SLE DA with increases in the mean score of body mass index (**Table 5**).

**Table 5.** The results of multivariate logistic regression analysis to evaluate the relationship of SLEDAI with HPLPII and personal characteristics.

**Independent variables**		**Odds Ratio**	**95% CI**	**P value**
Lifestyle	Total	0.210	0.068–0.643	0.006
	Physical Activity	1.919	0.874–4.210	0.104
	Stress management	0.283	0.072–1.113	0.071
	Health responsibility	0.449	0.165–1.225	0.118
	Nutrition	0.878	0.321–2.400	0.799
	Spiritual growth	0.381	0.118–1.233	0.107
	Interpersonal relationships	1.747	0.680–4.493	0.247

Age		1.002	0.972–1.033	0.907

Gender	Male*			
	Female	0.215	0.042–1.100	0.065

Educational level	Below diploma*			
	Diploma	1.715	0.758–3.878	0.195
	University	1.089	0.423–2.802	0.860

Cigarette smoking	Yes*			
	No	1.503	0.137–16.552	0.739

Duration of affliction by SLE (Month)		0.997	0.994–1.001	0.209

Body mass index		1.599 #	1.095–2.335	0.015

Body mass index	< 18.5 (Underweight)*			
	18.5–24.9 (Normal weight)	4.236	0.675–26.572	0.123
	25.0–29.9 (Overweight)	8.193	1.226–54.772	0.030
	30.0–34.9 (Class I obesity)	10.432	1.371–79.362	0.024
	35.0–39.9 (Class II obesity)	9.409	0.964–91.811	0.054

Positive family history of SLE	Yes			
	No	0.206	0.031–1.375	0.103

CI: Confidence interval;

# Trend effect;

* Reference category.

## DISCUSSION

This study aimed to evaluate the relationship of lifestyle with DA among patients with SLE. Findings showed that SLE DA had significant negative relationships with lifestyle and its stress management and spiritual growth dimensions and significant positive relationship with body mass index.

The total mean score of lifestyle was 2.49±0.30, indicating poor lifestyle among study participants. A study in Taiwan also showed that patients with SLE had poor lifestyle.^24^ Another study in Iran on patients with chronic diseases other than SLE (including hypertension, cancer, diabetes mellitus, and multiple sclerosis) also showed that the mean score of lifestyle was 2.47±0.41 among women and 2.49±0.37 among men.^34^

The lowest lifestyle dimensional mean score was related to the physical activity dimension (1.55±0.60). In line with this finding, a study on patients with SLE in Taiwan showed that the lowest lifestyle dimensional mean score was related to the physical activity dimension.^24^ Another study in Sweden showed that patients with SLE had lower physical activity capacity and less frequent exercise compared with population controls.^35^ Similarly, a study in Italy and a study in Iran showed that most patients with SLE had inadequate physical activity.^14^ A study in Iran on patients with chronic conditions also reported that the lowest lifestyle dimensional mean score was related to physical activity with a mean score of 1.95±0.63 among men and 1.84±0.66 among women.^34^ The low level of physical activity among patients with SLE may be due to SLE-related tissue injuries and psychological problems.^35^ Stress management was the second lowest-scored lifestyle dimension in the present study (score: 1.99±0.43). Stress management among patients with SLE is of great importance because physical and mental stress can be associated with disease relapse.^36^ However, a study in Iran on patients with chronic conditions showed that the mean score of the stress management dimension of lifestyle was 2.44±0.46 among men and 2.42±0.51 among women.^34^ Both these mean scores are greater than the mean score of stress management in the present study. This contradiction is attributable to the fact that patients in that study suffered from chronic conditions other than SLE, such as hypertension, cancer, diabetes mellitus, and multiple sclerosis.^34^ Patients with SLE have multi-systemic problems and hence, suffer from higher levels of stress.

The mean score of the spiritual growth dimension of lifestyle in the present study was 2.35±0.50, indicating poor spiritual growth. A study on patients with chronic conditions in Iran showed that the mean score of spiritual growth was 2.56±0.49 among men and 2.54±0.66 among women, which are in line with our findings.^34^ Two studies on patients with scleroderma, SLE, and malignant melanoma reported spiritual and mental needs as the most important needs of these patients.^37,38^ The debilitating conditions of patients with SLE in the active phase of the disease reduce their ability to perform activities related to spirituality and self-actualization. Similarly, a study in the United States reported the high prevalence of disability in performing valued life activities among patients with SLE.^39^

Our findings showed that the mean score of the interpersonal relationships dimension of lifestyle was 2.77±0.47. Similarly, a study on patients with chronic diseases in Iran showed that the mean score of this dimension was 2.64±0.47 among men and 2.69±0.52 among women.^34^ Patients with SLE have concerns over body image and role maintenance which trigger their embarrassment and withdrawal, and negatively affect their social functioning and relationships.^40^ Therefore, development of interpersonal relationships is an effective strategy to prevent disease activation among them, particularly among those with depression and severe SLE.^41^

Study findings showed that the mean score of the nutrition dimension of lifestyle was 2.87±0.40. This is in agreement with the findings of a study in Iran on patients with chronic diseases which reported that the mean score of the nutrition dimension among men and women was 2.79±0.51 and 2.77±0.50, respectively.^34^ Patients with SLE suffer from dyslipidaemias’ hypertension, and high value of glucose and hence, need to have a diet moderate in protein and high in vitamins, minerals, antioxidants, and polyunsaturated fatty acids.^25,42–44^ Nonetheless, a study in Iran reported that despite receiving glucocorticoids, only 24% of patients with SLE consumed a low-salt low-fat diet.^14^

The highest lifestyle dimensional mean score was 3.25±0.45 and was related to the health responsibility dimension. Contrarily, a study on patients with SLE in Iran reported that the mean score of health responsibility was 2.49±0.48 among men and 2.45±0.51 among women.^34^ This contradiction may be due to the fact that patients in the present study referred to the study setting to receive treatment and care-related services based on a predetermined time schedule, and were able to ask their health-related questions of a group of healthcare specialists (including rheumatology specialists, assistants, and residents) who spent a great deal of time on assessing patients’ problems and answering their questions. It is noteworthy that patients’ health responsibility is greatly affected by quality patient education and effective communication with healthcare providers.^45^

Study findings also showed that 67.5% of participants had active SLE. A cohort study on 1886 patients with SLE in Sweden showed that almost half of the participants maintained their original DA pattern during the three-year course of the study.^46^ Activation of SLE and subsequent wide use of corticosteroids and immunosuppressants severely damage different body organs which in turn aggravate afflicted patients’ conditions, and may finally result in death.^47^

Study findings also revealed a significant negative relationship between health-promoting lifestyle and SLE DA. In line with this finding, a systematic review showed that lifestyle had significant effects on SLE DA and noted that tobacco use increased the risk of skin changes and DA.^16^ A study in Japan also reported a significant relationship between lifestyle and well-being among patients with SLE.^25^ Moreover, our findings indicated that the stress management dimension of lifestyle had significant negative relationship with DA. In agreement with this finding, a study showed that stress can aggravate the clinical symptoms of SLE.^48^ Another study on patients with SLE in India reported that stress had significant relationship with lupus nephritis, so that patients with nephritis had higher levels of stress compared with those without nephritis.^45^ Similarly, a study in Korea showed that patients with higher levels of stress had greater SLE DA.^49^ In addition, we found that the spiritual growth dimension of lifestyle had significant negative relationship with SLE DA. Spirituality/religiosity is an important source of coping among patients with chronic conditions which can significantly reduce their distress.^50^

Study findings showed that around half of the participants were overweight or obese and there was a significant positive relationship between body mass index and SLE DA. Similarly, a former study reported the high prevalence of overweight and obesity among patients with SLE.^51^ A study in the United States also showed that high body mass index was an independent determinant of SLE DA.^52^

### Limitations

Among the limitations of the present study were non-random sampling and data collection through the self-report method. Patients used a wide range of medications, so that it was not possible to measure the relation of type of drug to disease activity. Therefore, it is suggested that in future studies, the relationship between the drugs categorisation used by patients and its relationship with disease activity should be investigated. It is also suggested that other underlying diseases of patients and organ involvement be considered in future.

## CONCLUSION

This study suggests that patients with SLE have poor lifestyle, most of them have active disease, and their DA has significant negative relationship with lifestyle and significant positive relationship with body mass index. Therefore, interventions are needed to promote lifestyle among these patients and thereby, reduce their DA. Improving physicians’ and nurses’ knowledge about SLE and lifestyle modification can also be an effective strategy for promoting health-related outcomes and reducing DA among patients with SLE. Other important strategies with potential effects on DA among these patients are interventions for promoting weight management, stress management, and spiritual growth.

## ABBREVIATIONS

CI: Confidence interval
DA: Disease Activity
HPLPII: Health-Promoting Lifestyle Profile II
SLE: Systemic lupus erythematosus
SLEDAI: Systemic Lupus Erythematosus Disease Activity Index
